# Pathological complete response after preoperative chemotherapy including FOLFOX plus bevacizumab for locally advanced rectal cancer: A case report and literature review

**DOI:** 10.1016/j.ijscr.2019.08.010

**Published:** 2019-08-17

**Authors:** Ryoichi Miyamoto, Kazunori Kikuchi, Atsushi Uchida, Masayoshi Ozawa, Naoki Sano, Sosuke Tadano, Satoshi Inagawa, Tatsuya Oda, Nobuhiro Ohkohchi

**Affiliations:** a Department of Gastroenterological Surgery, Tsukuba Medical Center Hospital, 1-3-1 Amakubo, Tsukuba, Ibaraki 305-8558, Japan; b Department of Pathology, Tsukuba Medical Center Hospital, 1-3-1 Amakubo, Tsukuba, Ibaraki 305-8558, Japan; c Department of Surgery, Division of Gastroenterological and Hepatobiliary Surgery and Organ Transplantation, University of Tsukuba, 1-1-1 Tennodai, Tsukuba, Ibaraki 305-8575, Japan

**Keywords:** Rectal cancer, Preoperative chemotherapy, Neoadjuvant chemotherapy, Bevacizumab, Pathological complete response

## Abstract

**Background:**

In Japan, the significance and efficacy of preoperative chemotherapy alone for locally advanced rectal cancer remain controversial. This case report presents the apparent effectiveness of preoperative FOLFOX plus bevacizumab as shown by pathological complete response (pCR). Additionally, we review the relevant literature and discuss the clinical management of locally advanced rectal cancer with preoperative chemotherapy.

**Case presentation:**

A 59-year-old male presented with severe constipation, bloody stool and a loss of 10% of his body weight over 3 months. Preoperative examination revealed locally advanced rectal cancer with extensive invasion of the bladder wall and enlarged regional lymph nodes. Thus, this lesion was assigned a preoperative classification of T4bN2bM0 stage IIIC according to the 8^th^ Union for International Cancer Control (UICC) guidelines. Therefore, the patient initially underwent an external loop colostomy of the transverse colon. Next, the patient received chemotherapy including FOLFOX plus bevacizumab. After 12 cycles of chemotherapy, the tumor size was markedly decreased, and all lymph node metastases had disappeared. Therefore, the patient underwent conventional resection of the rectum with D3 lymph node dissection and closure of the colostomy. Histopathological analysis of the resected specimen revealed that all lesions were fibrotic and devoid of any viable cancer cells. Thus, this lesion was assigned a final classification of ypT0N0M0 stage 0.

**Conclusions:**

We present the rare case of a patient with surgically resected locally advanced rectal cancer who demonstrated an impressive pCR with preoperative chemotherapy, which included FOLFOX plus bevacizumab.

## Highlights

• Preoperative chemotherapy alone for locally advanced rectal cancer remain unclear.
• Neoadjuvant chemotherapy was a promising treatment for unresectable rectal cancer.
• Earlier preoperative systemic chemotherapy was assumed to prevent the dissemination.

## 1 Introduction

In Western countries, the standard treatment for advanced stage rectal cancer is initially preoperative administration of fluorouracil-based chemoradiotherapy (CRT) [1,2]. Subsequently, neoadjuvant CRT followed by total mesorectal excision (TME) and postoperative systemic chemotherapy has been standard practice [3]. These therapeutic strategies have dramatically improved treatment results for rectal cancer, with the local recurrence rate reported to be 10% [4,5]. However, radiotherapy (RT) is well known to cause several late complications, such as urinary and sexual dysfunction [6], intestinal and defecation problems [7] and secondary carcinogenesis [8]. Furthermore, there remains a significant occurrence of distant metastasis [[9], [10], [11]].

In contrast, preoperative RT for primary rectal cancer is nontraditional, and radical surgery, including lateral lymph node dissection followed by postoperative systemic chemotherapy, is the standard treatment in Japan [12]. The current 5-year local recurrence rate of 6% is almost acceptable [13]. However, the greatest problem is the high rate of distant metastasis after curative resection [14]. Therefore, we assumed that suppression of hematogenous distant metastasis from primary rectal cancer might be the fastest route to an improved survival rate.

Previous studies reported that patients with advanced stage rectal cancer who were initially treated with neoadjuvant chemotherapy (NAC) alone, including fluorouracil, leucovorin and oxaliplatin (FOLFOX) plus bevacizumab, demonstrated an impressive pathological complete response (pCR) rate of 25–35% and favorable outcomes [10,15]. However, the significance and efficacy of the therapeutic strategy involving preoperative chemotherapy alone for locally advanced rectal cancer remain controversial.

In the present case report, we present the apparent effectiveness of preoperative chemotherapy, including FOLFOX plus bevacizumab and radical surgery, as shown by pCR. Additionally, we review the relevant literature and discuss the clinical management, including preoperative chemotherapy, for locally advanced rectal cancer. This case report was presented in line with the SCARE criteria [16].

## 2 Presentation of case

**Fig. 1:**
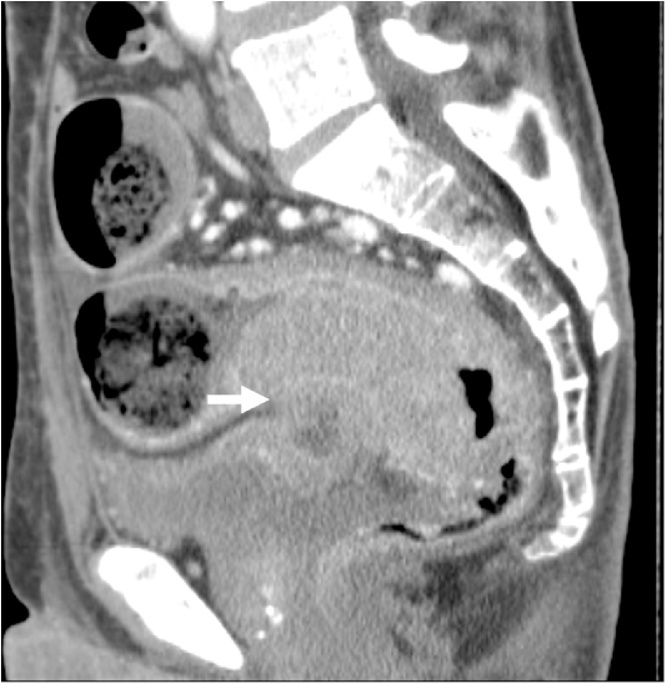
Abdominal CT revealed that the wall thickness of the rectum showed extensive invasion of the bladder wall (arrow). CT, computed tomography.

**Fig. 2:**
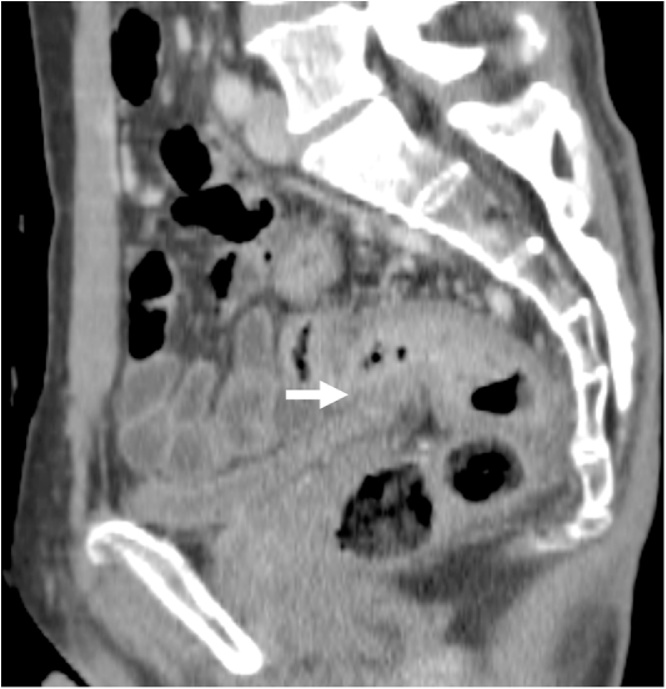
Abdominal CT after chemotherapy revealed that the tumor size was markedly decreased and extensive invasion between the rectal tumor and bladder wall was not observed (arrow). CT, computed tomography.

**Fig. 3:**
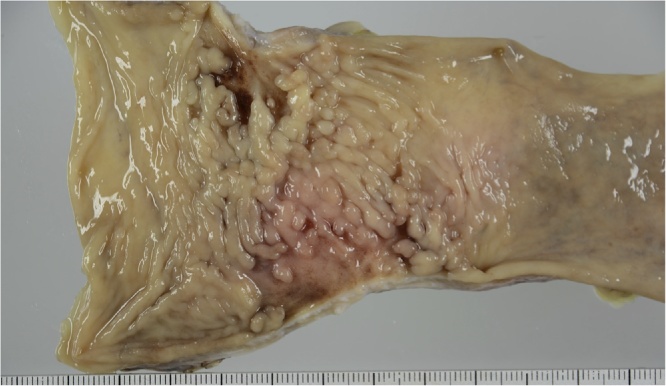
Resected specimen from the anterior resection showing a fibrous scar in the rectum.

**Fig. 4:**
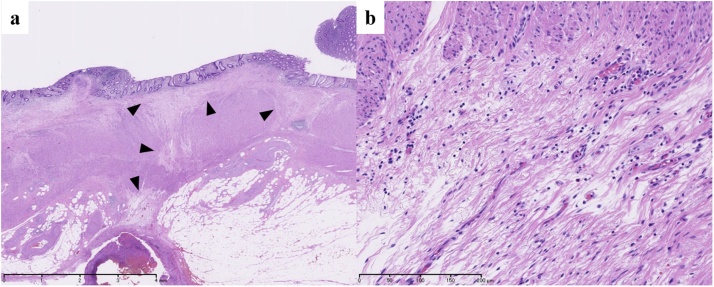
Histological analysis of the operative specimen, showing the absence of viable cancer cells and the presence of fibrous scarring within the partially reepithelialized rectal wall (arrowhead). Hematoxylin and eosin staining, ×12.5 (a); hematoxylin and eosin staining, ×200 (b).

A 59-year-old male presented with severe constipation, bloody stool and a loss of 10% of his body weight over 3 months. The patient’s medical history included diabetes mellitus and angina pectoris. His abdomen was soft and exhibited no tenderness on examination. Laboratory findings revealed a hemoglobin concentration of 14.7 g/dL. Serum carcinoembryonic antigen and carbohydrate antigen 19-9 levels were 130.7 ng/mL and 67.3 U/mL, respectively. Colonoscopy revealed a tumor with ulceration in the rectum (15 cm above the anal verge). Abdominal computed tomography revealed that the wall thickness of the rectum showed extensive invasion of the bladder wall and enlarged regional lymph nodes (Fig. 1). Distant organ metastasis, including liver metastasis and peritoneal dissemination, was not observed on the preoperative radiological findings. Thus, the lesion was assigned a preoperative classification of T4bN2bM0 stage IIIC according to the 8th Union for International Cancer Control (UICC) guidelines. Therefore, the patient initially underwent external loop colostomy of the transverse colon. Next, the patient received chemotherapy including FOLFOX plus bevacizumab. After 12 cycles of chemotherapy, the tumor size was markedly decreased, and all lymph node metastases had disappeared. Furthermore, extensive invasion between the rectal tumor and bladder wall was not observed (Fig. 2). Subsequently, the patient underwent conventional resection of the rectum with D3 lymph node dissection and closure of the colostomy. Histologically, a fibrous scar was observed at the level of the previously documented tumor, and histological examination of the surgical specimen did not reveal any viable cancer cells in the rectal wall or in the mesorectum (Fig. 3, Fig. 4a, b). Thus, the lesion was assigned a final classification of ypT0N0M0 stage 0. The patient’s postoperative recovery was uneventful, and he was discharged from the hospital after 11 days. The patient did not exhibit recurrence during the 36-month follow-up period. The ethics committee of the Tsukuba Medical Center Hospital approved this study (#2019-010). Informed consent was obtained for the publication of this case from the patient concerned.

## 3 Discussion

In the present case report, we present the rare case of a patient with surgically resected locally advanced rectal cancer who demonstrated an impressive pCR after preoperative chemotherapy, including FOLFOX plus bevacizumab.

In the treatment strategy for locally advanced rectal cancer, we assumed that the major paradigm had shifted toward the use of TME and neoadjuvant therapy instead of adjuvant therapy, which led to significant advances in the local control and overall survival of these patients. Therefore, we assumed that earlier preoperative systemic chemotherapy to prevent the dissemination of micrometastases improved survival by reducing distant metastasis.

Recently, combinations of doublet/triplet chemotherapies have markedly improved the response rate and prognosis of unresectable and recurrent colorectal cancer. Oxaliplatin-based combination chemotherapy, including FOLFOX, improves the survival of colorectal cancer patients in metastatic and adjuvant circumstances [17]. In patients with advanced rectal cancer, NAC alone has been increasingly used to reduce the size of tumors and render initially unresectable tumors potentially resectable [18]. In fact, our case report shows that the tumor size was markedly decreased and that all lymph node metastases had disappeared after preoperative chemotherapy, which included FOLFOX plus bevacizumab. Schrag et al. treated patients with clinical stage II-III rectal cancer with neoadjuvant FOLFOX-based chemotherapy (6 cycles of FOLFOX in which bevacizumab was included in the first 4 cycles) followed by TME [10]. They reported a pCR rate of 27%, an R0 resection rate of 100%, and no local recurrence during a median follow-up period of 18.2 months. Similarly, a review from Memorial Sloan Kettering Cancer Center of 20 patients with colorectal cancer who were treated initially with FOLFOX +/– bevacizumab also demonstrated an impressive pCR rate of 35% [15]. In Japan, a multicenter phase II trial of NAC with XELOX plus bevacizumab for locally advanced rectal cancer reported a satisfactory short-term outcome with a completion rate of 84.4% and a pCR rate of 13.3% [9].

NAC alone in advanced rectal cancer presents several difficulties that must be addressed. First, the rate of postsurgical complications was not negligible. In particular, the preoperative use of bevacizumab is of concern to surgeons because bevacizumab-related delayed wound healing might increase postoperative complications, including wound infection, anastomotic leakage and rectal perforation [19]. Therefore, a 5- to 8-week interval between the last administration of bevacizumab and elective surgery is recommended and widely accepted [20]. In our case report, conversion surgery was performed at 5 weeks after the last bevacizumab-containing chemotherapy.

## 4 Conclusion

We present the rare case of a patient with surgically resected locally advanced rectal cancer who demonstrated an impressive pCR with preoperative chemotherapy, including FOLFOX plus bevacizumab. We consider NAC to be a promising preoperative treatment for locally advanced rectal cancer instead of neoadjuvant CRT. However, there are no data about whether NAC with or without concurrent radiotherapy is effective against advanced rectal cancer, and further studies are needed. Whether bevacizumab should be added in neoadjuvant settings requires further careful investigation because of its serious perioperative complications.

## Sources of funding

This research did not receive any specific grant from funding agencies in the public, commercial, or not-for-profit sectors.

## Ethical approval

The ethics committee of the Tsukuba Medical Center Hospital approved this study (#2019-010).

Informed consent was obtained for the publication of this case from the patient concerned.

## Consent

Informed consent was obtained for the publication of this case from the patient concerned.

## Author contribution

All authors participated in the treatment of this case.

Miyamoto R. wrote this paper.

All authors read and approved the final manuscript

## Registration of research studies

We have completed registration of research studies (#researchregistry4975).

## Guarantor

Miyamoto Ryoichi

## Provenance and peer review

Not commissioned, externally peer-reviewed

## Declaration of Competing Interest

There are no potential conflicts of interest to disclose.
